# 

**Published:** 1887-06-04

**Authors:** 


					i!
C) | '
[THIRTY-TWO PAGES, INCLUSIVE OF EIGHT EXTRA.]
No. 36, Vol. II. W W a PRICE ONE PENNY WEEKLY.
June 4th, 1887.
wir bub n a
PftT Annnm. fis. fid .-nnat froo
_ ,m, ipp?wi igKz\ \a . u? .rer Annum, t>s. ed., post free.
'^ClstcrtMi nt\C^ ' \1\ fUl /JT^ Monthly Parts. 6d.; post free, 7id.
?* a Xc*vS?S r?St ? ^ |Sk Ditto per Ann., 7s. 6d., post free.
an Institution, .-jfamilg, an& $arocfjtal journal
hospitals. 3st>Iums. & all itlgrimcs for ttjc gate of tf)c $tcfe. Criticism & flrtos.

				

